# Lymphocytic Myocarditis in Children with Parvovirus B19 Infection: Pathological and Molecular Insights

**DOI:** 10.3390/biomedicines12081909

**Published:** 2024-08-20

**Authors:** Lisann Pelzl, Sabrina Mantino, Martina Sauter, Tatiana Manuylova, Ulrich Vogel, Karin Klingel

**Affiliations:** Cardiopathology, Institute for Pathology and Neuropathology, University Hospital of Tuebingen, 72076 Tuebingen, Germany; lisann.pelzl@med.uni-tuebingen.de (L.P.); smantino@dna-diagnostik.hamburg (S.M.); martina.sauter@med.uni-tuebingen.de (M.S.); tatiana.manuylova@med.uni-tuebingen.de (T.M.); ulrich.vogel@med.uni-tuebingen.de (U.V.)

**Keywords:** parvovirus B19 (B19V), viral myocarditis, children, endomyocardial biopsies, cardiomyopathy

## Abstract

Background: This study aims to evaluate the role of parvovirus B19 (B19V) in the pathogenesis of myocarditis in a paediatric population, including post-mortem samples from two children. Methods: From 2004 to 2023, endomyocardial biopsies (EMBs) from children under 16 years of age were analyzed using histology, immunohistochemistry, and molecular pathology. A total of 306 children with acute and 1060 children with chronic lymphocytic myocarditis were identified. Results: B19V infection was more frequent in acute myocarditis than in chronic myocarditis (43% vs. 14%), with higher viral loads in acute cases regardless of age. The most prominent cardiac CD3+ T cell infiltration was noted in children < 2 years, correlating with high cardiac B19V loads. In two male infants who died from B19V infection, B19V DNA was localized in the endothelial cells of multiple organs using in situ hybridization. Virus replication was found in the endothelial cells of small cardiac arterioles and venules but not in capillaries. B19V DNA/mRNA was also detected in immune cells, especially in the spleen and lymph nodes, revealing virus replication in B lymphocytes. Conclusions: B19V can induce severe lymphocytic myocarditis, especially in young children. The simultaneous histopathological and molecular assessment of EMBs is important for early diagnosis of viral myocarditis, preventing severe disease, and ensuring appropriate therapy.

## 1. Introduction

Parvovirus B19 (B19V) is a small single-stranded DNA virus of the Erythrovirus genus, which is known to induce acute and chronic lymphocytic myocarditis in adults and children [1,2,3,4,5]. Viral myocarditis is rare in children, and nowadays, it is often caused by B19V [6]. Acute B19V myocarditis in children is mostly associated with a primary B19V infection being characterized by systemic viremia with viral concentrations of up to 10^11^–10^13^ virus copies/mL in blood 6 to 10 days after viral transmission. By day 10, a loss of erythroid precursors is noted in the bone marrow. When exanthema occurs after an average of 14 days, blood and saliva still contain approximately 10^4^–10^8^ virus copies/mL. In children, the virus is mostly cleared from the blood within 3–4 weeks [7]. However, B19V DNA persistence has been detected in various tissues in low copy numbers, mostly not associated with obvious clinical disease [8,9]. B19V infection confers lifelong immunity. Infection rates increase proportionally with age and are around 60–70% in adults. Children with B19V infection present with a range of symptoms, from paucisymptomatic illness to acute or chronic anemias, arrhythmias, sinus tachycardia, myocarditis, acute heart failure, and sudden cardiac death [2,10,11].

Unlike enteroviruses, which induce lymphocytic myocarditis following the infection of cardiomyocytes, B19V infects endothelial cells in the myocardium [4]. The interaction between B19V and the human immune system is complex and multifaceted. In addition to the heart, B19V also infects various other tissues and organs, which is based on the distribution of its most important cellular receptor, globoside (Gb4) [12]. B19V replication occurs in erythroid progenitor cells, placental trophoblast, synovium, and endothelium, which is relevant for understanding the pathogenesis of B19V infections [13]. Infection of endothelial cells in the heart has been associated with impaired cardiac microcirculation, secondary myocyte necrosis, and inflammation in adult patients [5]. Whether the consequences of B19V infection in children are comparable to those observed in adults is not yet clear. Therefore, we investigated the role of B19V in the pathogenesis of myocarditis in a paediatric population by evaluating its prevalence in a large cohort of children with endomyocardial biopsy (EMB)-proven acute and chronic lymphocytic myocarditis and by correlating molecular and immunopathological findings in two age groups (≤ or >2 years). We found high copy numbers of B19V in EMB, especially in very young children, associated with severe acute lymphocytic myocarditis. To underline the detrimental effects of B19V infection in very young children, we demonstrate the patterns of infection and inflammation in organs from two infants who died of B19V myocarditis.

## 2. Materials and Methods

### 2.1. Patients

We screened our database of diagnostic EMBs for children with clinically suspected myocarditis between 2004 and 2023. Criteria for the inclusion were the age of the patients (<16 years) and a diagnosis of acute lymphocytic or healing/chronic lymphocytic myocarditis. Patients with a history of autoimmune disease, solid organ or hematopoietic stem cell transplantation, heart malformations, and associated surgery were excluded from this study. EMBs were analyzed via histology, immunohistology, and molecular pathology. Acute lymphocytic myocarditis was defined by the presence of myocyte necrosis and lymphocytic inflammation, with lymphocytes >25/mm^2^ and increased numbers of macrophages. Healing/chronic lymphocytic myocarditis (in the paper given as chronic myocarditis) was diagnosed in the absence of myocyte necrosis, but in the presence of lymphocytes ≥ 7/mm^2^, increased numbers of macrophages, and focal or diffuse fibrosis, according to the Dallas criteria and the position statement of the European Society of Cardiology Working Group on Myocardial and Pericardial Diseases [11,14]. Autopsies were performed on two very young male children (12 and 17 months old) who died of acute B19 infection. The first patient (patient A, 12 months old) had a cough and fever. Eight days later, his general condition deteriorated. He developed metabolic acidosis with hyperkalemia, elevated levels of transaminases and creatine kinase, and massive global heart failure. He died 15 days later. The second patient (patient B, 17 months old) was infected by his twin brother, who showed no signs of myocarditis. He developed massive cerebral edema, multifunctional intestinal failure, and cardiogenic circulatory shock with dilated cardiomyopathy. We obtained different organs, which were fixed in 4% formaldehyde, embedded in paraffin, and investigated via histology, immunohistology, and quantitative PCR; radioactive and non-radioactive in situ hybridization was performed for the localization of B19V nucleic acids in the tissue.

### 2.2. Histology and Immunohistochemistry in EMB

EMBs were fixed in 4% phosphate-buffered formalin and embedded in paraffin. Four µm-thick tissue sections were stained with haematoxylin–eosin, Masson’s Trichrome, and Giemsa and examined using light microscopy. For immunohistological detection of cardiac immune cells, a monoclonal rabbit anti-CD3 antibody (clone SP7, 1:500, Novocastra Laboratories, Newcastle upon Tyne, UK), a monoclonal mouse anti-human CD68 antibody (clone PG-M1, 1:50) and a monoclonal mouse anti-human HLA-DR alpha-chain antibody (clone TAL.1B5, 1:50), both from DAKO, Hamburg, Germany, were used. Immunohistochemical analysis was performed on an automated immunostainer according to the manufacturer’s protocol (Benchmark; Ventana Medical Systems, Tucson, AZ, USA) using the ultraView detection system (Ventana) and diaminobenzidine as the substrate. Tissue sections were counterstained with haematoxylin.

### 2.3. PCR

Detection of viral genomes via nested PCR was performed on heart tissue and blood samples as previously described [15]. In brief, nested PCR/reverse transcription–PCR was performed on RNA extracted from EMBs to detect enteroviruses (EV, including coxsackieviruses and echoviruses) and on DNA to detect B19V, adenoviruses (ADV), and human herpesvirus 6 and 7 (HHV6 and HHV7), human cytomegalovirus (HCMV), and Epstein–Barr virus (EBV). As a control for the successful extraction of DNA and RNA from heart muscle tissue, oligonucleotide sequences were chosen from the DNA sequence of the glyceraldehyde 3-phosphate dehydrogenase gene. The specificity of amplification products was shown via Sanger Sequencing using a CEQ 2000XL_X (AB Sciex, Darmstadt, Germany), as previously described [5].

### 2.4. Quantification of B19V DNA via PCR

B19V DNA was quantified using a Rotorgene system (Qiagen, Hilden, Germany) in all patient samples that were positive in the previous PCR [1].

### 2.5. In Situ Hybridization (ISH)

In situ detection of B19V DNA was performed on dewaxed 5 μm tissue sections using a previously described protocol [5]. The hybridization mixture contained a 35S-labeled RNA antisense B19V probe, which was transcribed in vitro from the 2.5 kb HindIII/Eco RI fragment of cloned B19V [1]. After the post-hybridization washing procedures, the tissue slide preparations were autoradiographed and stained with haematoxylin and eosin. Negative control RNA probes were synthesized from a non-recombinant transcription vector bluescript KS- and from plasmid pCVB3-R1, which provide EV-specific RNA probes [5]. Double-labeling experiments were performed via immunohistochemical staining of T and B lymphocytes and subsequent radioactive in situ hybridization, as previously published for enteroviruses [16].

In addition, non-radioactive in situ hybridization experiments were performed. To prove virus replication by detecting B19V DNA and mRNA, heart tissue sections were hybridized using specific sense and antisense probes for B19V (ACD, Newark, CA, USA), followed by the RNAscope 2.5 HD Detection Kit Red from ACD (Newark, CA, USA), according to the manufacturer’s protocol.

### 2.6. Statistical Analyses

Group comparisons were performed using the Kolmogorov–Smirnov test. Correlations were performed with the Pearson test. All analyses were two-tailed, and a *p*-value of <0.05 was defined to indicate a statistically significant difference.

### 2.7. Ethic Statement

This study was conducted in accordance with the Declaration of Helsinki. Retrospective data collection and anonymized analysis of the patients were conducted in accordance with local government law without the requirement for informed consent. The study protocol was approved by the Institutional Review Board of the University of Tuebingen (411/2021BO2).

## 3. Results

### 3.1. B19V DNA Is Detected in EMB from Children with Acute or Chronic Lymphocytic Myocarditis

**Figure 1:**
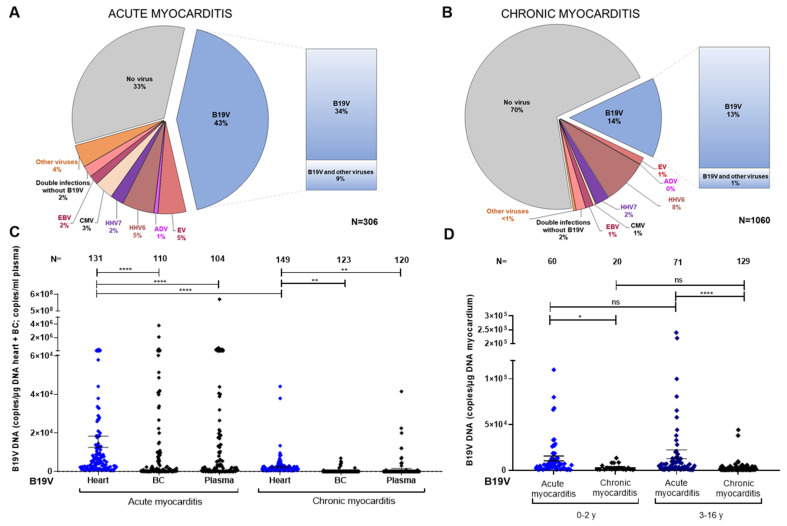
Detection of viral DNA/RNA in children with histological evidence of myocarditis. (**A**) PCR results from the heart tissue of 306 patients with histologically proven acute myocarditis. (**B**) PCR results from the heart tissue of 1060 patients with chronic myocarditis. (**C**) Comparison of viral copy numbers in the heart, buffy coat (BC), and plasma of all children with acute and chronic myocarditis. (**D**) Comparison of viral copy numbers in heart samples from two age groups (0–2 years and 3–16 years) with acute and chronic myocarditis. B19V viral load is presented as the number of copies per µg of DNA (heart or BC) and per ml of plasma. Data are presented as mean ± SEM; ns, not significant; * *p* < 0.05, ** *p* < 0.01, and **** *p* < 0.0001.

**Figure 2:**
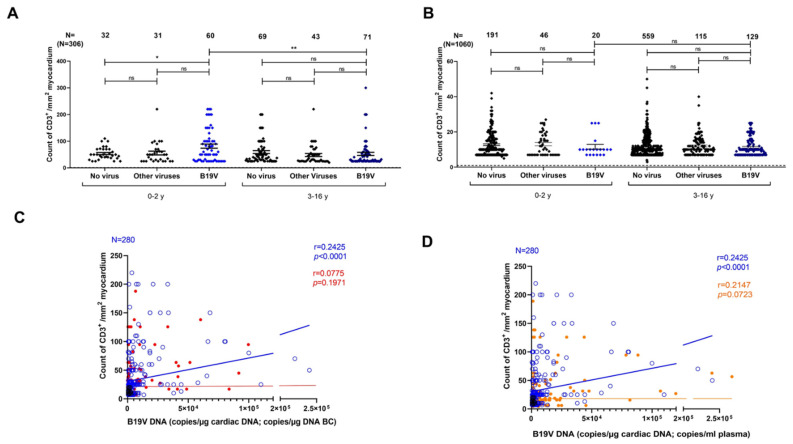
Correlation of CD3+ T cell infiltration and B19V DNA copies in children with acute and chronic myocarditis. (**A**) Comparison of CD3+ cell count in acute myocarditis without infection, other virus infections, and B19V infection between two age groups (0–2 years and 3–16 years). (**B**) Comparison of CD3+ cell count in chronic myocarditis without infection, other virus infections, and B19V infection between two age groups (0–2 years and 3–16 years). (**C**) Correlation of CD3+ T cell infiltration with viral DNA load in the myocardium (blue) and BC (red). (**D**) Correlation of CD3+ T cell infiltration with viral load in the myocardium (blue) and plasma (red). CD3+ cell count is presented as the number of cells per mm^2^. B19V DNA load is given as the number of copies per µg of DNA (heart or BC) and per ml of plasma. Data are presented as mean ± SEM; ns, not significant; * *p* < 0.05, and ** *p* < 0.01.

Between 2004 and 2023, lymphocytic myocarditis was diagnosed in the EMB of 1366 paediatric patients. Of these, 306 cases had acute lymphocytic myocarditis (147 females and 159 males) and 1060 cases had chronic lymphocytic myocarditis (442 females and 618 males). B19V DNA was detected in 131 (43%) of the EMBs revealing acute myocarditis (Figure 1A and Figure 2A). B19V DNA alone was found in 103 (34%) of the cases, while 28 cases (9%) displayed dual or multiple infections together with HHV6, HHV7, CMV, EBV, or EV. Viral nucleic acids other than B19V DNA were detected in 74 (24%) of the EMBs (Figure 1A and Figure 2A).

In patients with chronic lymphocytic myocarditis, B19V DNA was found in EMB from 149 (14%) cases. The presence of cardiac B19V DNA in the absence of other viral nucleic acids was still comparatively high in 138 (13%) cases. B19V DNA was detected together with other viral nucleic acids in EMB from 11 (1%) cases. Other cardiotropic viral nucleic acids, in the absence of B19V DNA, were detected in 161 (16%) EMBs (Figure 1B and Figure 2B).

Detection of viral DNA/RNA in blood was performed on 517 children with acute and chronic myocarditis. B19V DNA was found in the heart but not in the BC of 43 (39.1%) patients with acute myocarditis. In children with positive B19V DNA in EMB and chronic myocarditis, B19V DNA was not detected in the BC of 115 (93.5%) patients. Next, quantitative PCR was performed on all B19V DNA-positive EMBs with acute and chronic lymphocytic myocarditis to determine the individual viral load in EMB, buffy coat (BC), and plasma (Figure 1C). In patients with acute myocarditis (N = 306), the viral load in the myocardium was significantly higher compared to chronic lymphocytic myocarditis (N = 1060) (mean viral DNA copy number ± SEM: 15,395 ± 2901 copies/µg cardiac DNA vs. 2386 ± 407.8 copies/µg cardiac DNA, Figure 1C). Interestingly, the viral load in acute myocarditis was lower in heart samples compared to BC or plasma (mean viral DNA copy number ± SEM: 15,395 ± 2901 copies/µg cardiac DNA vs. 67726 ± 39318 copies/µg BC DNA vs. 5,508,777 ± 5,480,502 copies/mL plasma DNA, *p* < 0.0001, Figure 1C). This high range reflects that the timing of B19V DNA measurement in the blood is critical with regard to the onset of acute infection. As expected, we observed that the increase in B19V DNA in the heart occurs with a delay following high infection rates in the blood.

In addition, we analyzed the mean cardiac B19V DNA copy number in two age groups for acute and chronic lymphocytic myocarditis. In samples from 0–2-year-old patients with acute myocarditis (N = 60), the viral load was significantly higher compared to those with chronic myocarditis (N = 20) (mean viral DNA copy number ± SEM: 12,911 ± 2680 copies/µg cardiac DNA vs. 3279 ± 793.2 copies/µg cardiac DNA, *p* = 0.0001). Similarly, samples from 3–16-year-old patients with acute myocarditis (N = 71) showed significantly higher viral copy numbers compared to those with chronic myocarditis (N = 129) (mean viral DNA copy number ± SEM: 17,494 ± 4856 copies/µg cardiac DNA vs. 2247 ± 454.4 copies/µg cardiac DNA, *p* = 0.0001, Figure 1D). Comparing cases of acute lymphocytic myocarditis in children up to 2 years of age with those aged 3–16 years, there was no significant difference in cardiac B19V DNA copy numbers (mean viral cardiac DNA copy number ± SEM: 12,911 ± 2680 vs. 17,494 ± 4856, ns; Figure 1D).

### 3.2. Correlation of Immune Cell Infiltrates in Myocardium with B19V Infection in the Heart and Blood

The extent of cardiac CD3+ T cell infiltration was measured in two age groups—0–2 years and over 3 years—with acute infectious myocarditis (positive for nucleic acids from B19V or other viruses) and non-infectious myocarditis (Figure 2A). In 0–2-year-old children, the amount of CD3+ T cell infiltration in EMB was higher in the cardiac B19V DNA-positive group compared to the group infected with other viruses (mean CD3+ T cell infiltration number ± SEM: 81.45 ± 7.80 vs. 55.68 ± 7.12, *p* < 0.0001) or the non-viral group (53.66 ± 4.05, *p* < 0.0001, Figure 2A). In children older than three years, no significant difference was observed between the three groups. Importantly, we found that the amount of infiltrating CD3+ T cells was significantly increased in the group of very young children (0–2 years) with B19V-mediated acute myocarditis compared to patients older than 3 years with B19V-mediated acute myocarditis (mean CD3+ T cell infiltration number ± SEM for 0–2 years vs. 3–16 years: 81.45 ± 7.80 vs. 52.46 ± 5.93, *p* < 0.01, respectively; Figure 2A).

In chronic myocarditis, cardiac CD3+ T cell infiltration did not significantly differ between the age groups 0–2 years and 3–16 years, neither in the group with non-infectious myocarditis nor in both groups with infectious myocarditis (mean CD3+ T cell infiltration number ± SEM: 0–2 years vs. 3–16 years: 11.60 ± 1.36 vs. 11.38 ± 0.46; ns, respectively; Figure 2B).

As demonstrated in Figure 2C,D, in acute and chronic myocarditis patients, CD3+ T cell infiltration positively correlated with cardiac B19V DNA copy number (blue) (r = 0.2425, *p* < 0.0001) but not with the viral DNA copy number in BC (red) (BC: r = 0.0775, ns; Figure 2C) and not with the viral DNA copy number in plasma (red) (r = 0.2147, ns; Figure 2D).

### 3.3. High Viral Load in Multiple Organs in Children with Acute B19V Infection

**Figure 3:**
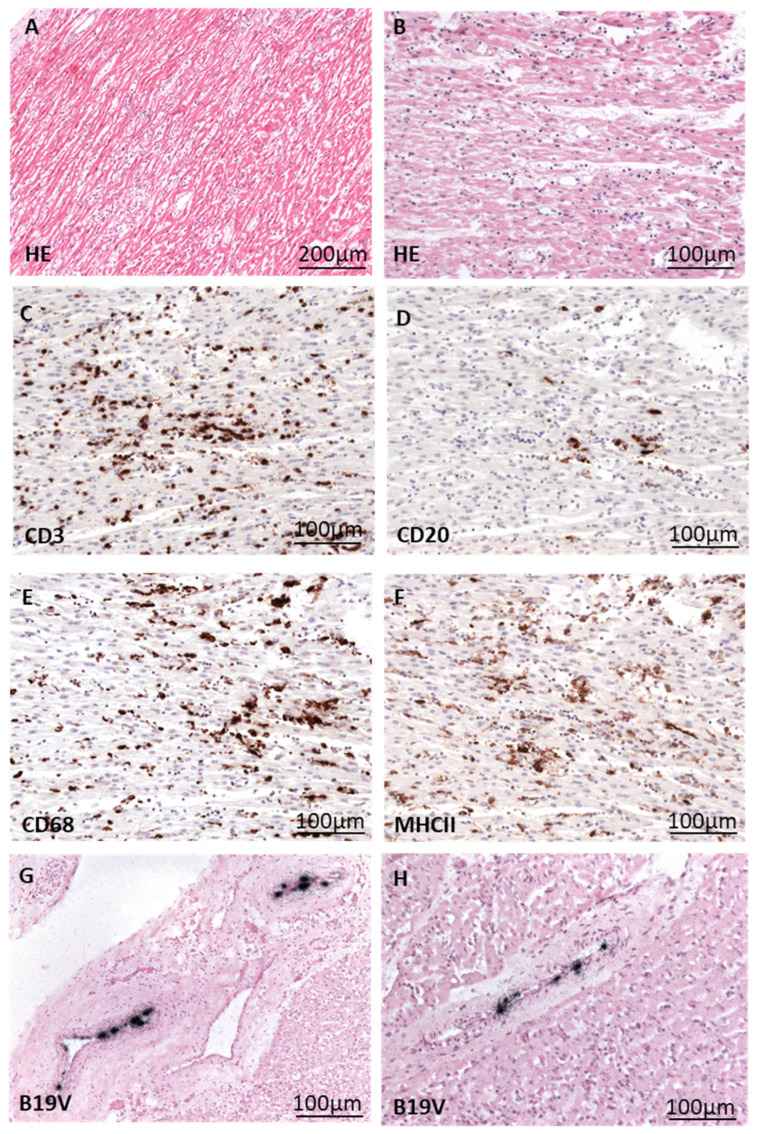
Histological/immunohistological presentation of fatal myocarditis in a 12-month-old patient (**A**) with cardiac B19V infection. (**A**,**B**) HE staining of heart tissue shows acute myocarditis characterized by myocyte necrosis and extensive inflammatory infiltrate. (**C**–**F**) Immunohistochemical staining (brown cells) reveals the presence of many CD3+ T cells (**C**), some CD20+ B cells (**D**), and numerous CD68+ macrophages (**E**), with many of them expressing MHCII (**F**). (**G**,**H**) Detection of B19V DNA (black signals) via radioactive ISH in endothelial cells of cardiac vessels.

To investigate B19V infection in small children in more detail, two male patients who died in the course of systemic B19V infection were investigated following an autopsy. In patient A, besides myocyte necrosis, an extensive infiltration with mononuclear immune cells was detected via histology and immunohistology. Whereas numerous CD3+ T lymphocytes and CD68+ macrophages with expression of MHCII were noted, less numbers of CD20+ B cells were observed (Figure 3).

**Figure 4:**
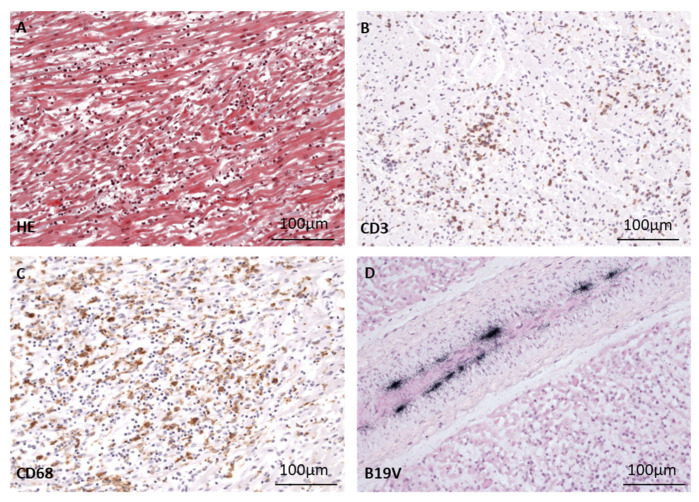
Morphological presentation of fatal myocarditis in a 17-month-old patient B after cardiac B19V infection. (**A**) Masson’s trichrome staining of heart tissue shows acute myocarditis with myocyte necrosis, many CD3+ T cells (**B**) and CD68+ macrophages (**C**) comparable to findings in patient A. (**D**) Detection of B19V DNA (black signals) via radioactive ISH in the endothelium of a cardiac vessel.

Patient B showed similar patterns of immune cell infiltration in the myocardium (Figure 4).

In both patients, B19V DNA was clearly detected in the endothelial cells of cardiac arterioles and venules via radioactive ISH (Figure 3G,H and Figure 4D). It is important to note that in both patients, B19V DNA was not detected in the endothelial cells of capillaries.

In addition, individual viral loads of different organs were determined (Table 1).

Both patients showed high viral copy numbers in their hearts—between 7.2 × 10^3^ (patient B) and 5.88 × 10^4^ copies/µg isolated DNA (patient A). In addition, B19V DNA was detected in the lungs, lymph nodes, spleen, liver, and kidneys of both children and in the brain tissue of patient A. In particular, high viral loads were noted in lymphatic tissues, ranging from 3.7 × 10^5^ (patient B) to 7.3 × 10^5^ copies/µg isolated DNA (patient A), confirming an acute systemic B19V infection in both children.

### 3.4. Localization of B19V DNA in Various Organs via ISH

**Figure 5:**
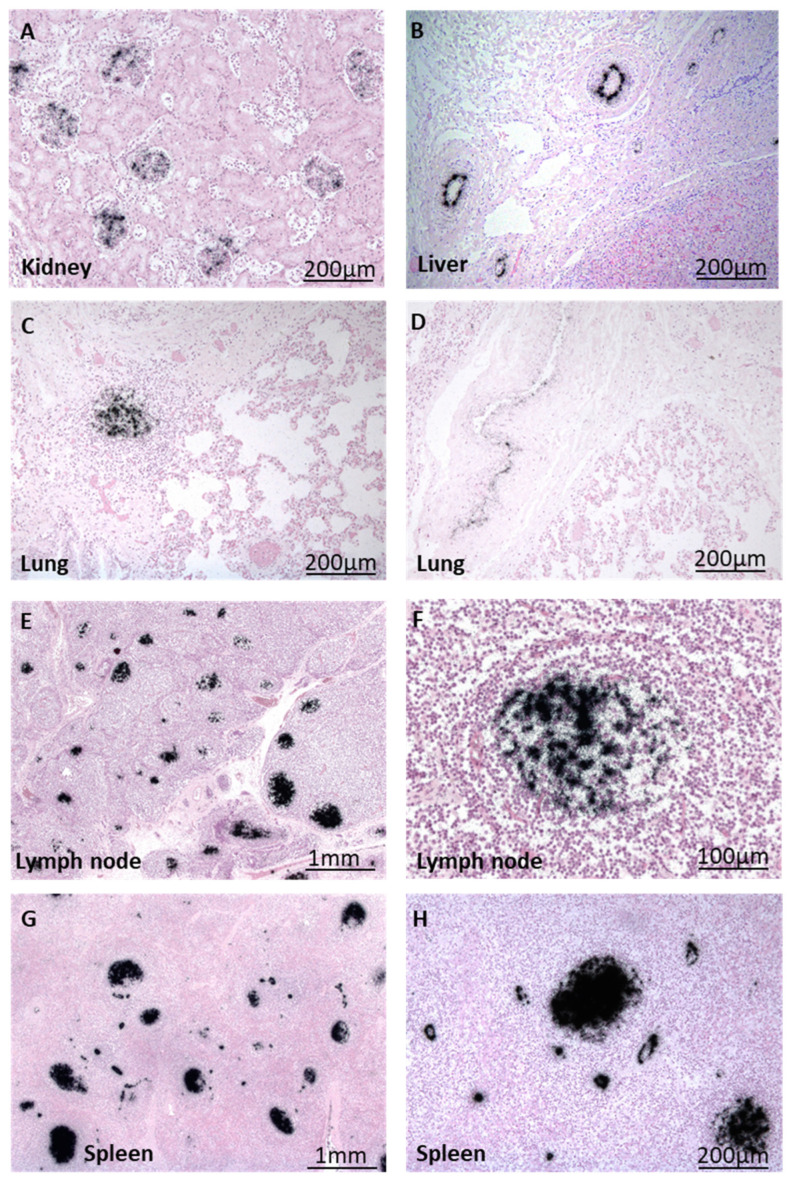
Visualization of B19V DNA (black) via radioactive ISH in different organs of patient B. (**A**) Localization of B19V DNA within kidney glomeruli and (**B**) arterioles of liver tissue. (**C**,**D**) B19V-positive immune cells and arterioles in the lung. (**E**) B19V DNA is present in numerous immune cells of lymph nodes and endothelial cells, with a close-up shown in (**F**). (**G**) B19V DNA-positive follicles and vessels in splenic tissue, with a close-up shown in (**H**).

Surprisingly, in contrast to the heart, other organs did not reveal inflammation in both children. However, B19V DNA was detected in many organs of both patients via ISH, reflecting the acute systemic B19V infection, as already shown using PCR. The ISH results for patient A are presented in Figure 5.

Radioactive ISH revealed viral DNA in the glomeruli of the kidney (Figure 5A), in lymphocytic infiltrates and the endothelium of small arterioles and venules in the lung (Figure 5C,D), in follicles of lymph nodes (Figure 5E,G (higher magnification)), and in the spleen (Figure 5F,H (higher magnification)) of this patient. B19V DNA was also detected in the endothelia of arterioles in the liver (Figure 5B).

### 3.5. Identification of B19V-Infected Splenic Immune Cells via Double Labeling

**Figure 6:**
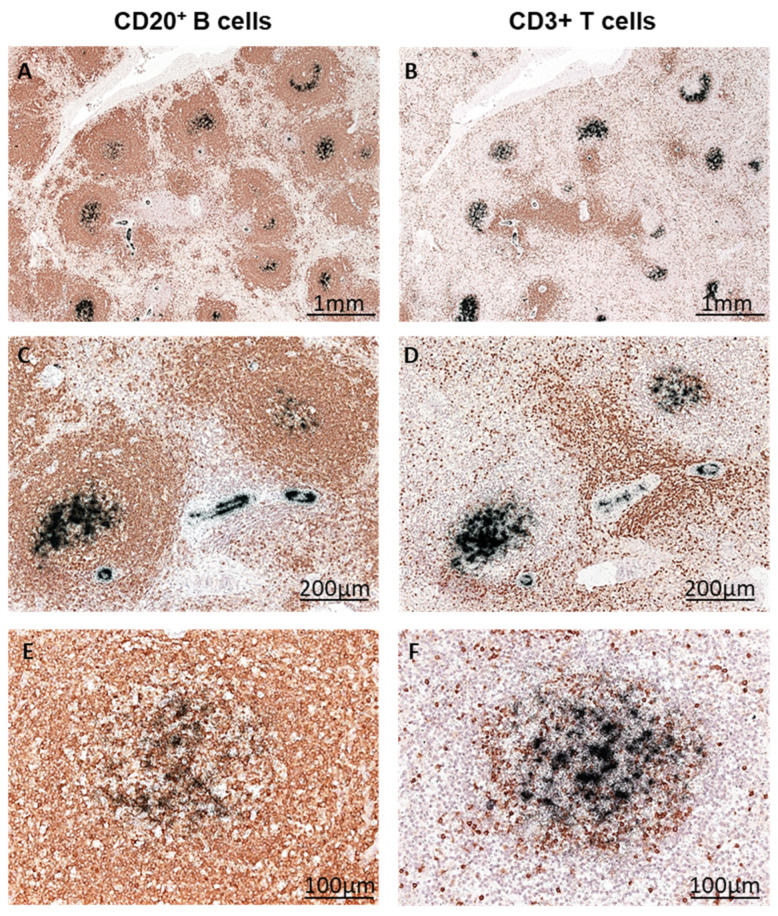
B19V replication in B cells of the spleen. Splenic tissue from patient B was immunohistochemically stained for CD20+ B lymphocytes (**A**,**C**,**E**) and CD3+ T lymphocytes (**B**,**D**,**F**) (visualized in brown). Consecutive radioactive ISH clearly shows the localization of B19V DNA in B cells (black signal) at different magnifications (**A**–**E**).

In splenic tissue sections, immunohistochemical staining of CD20+ B-cells and CD3+ T cells and consecutive radioactive in situ hybridization revealed the presence of B19V DNA in CD20+ B lymphocytes (Figure 6A,C,E different magnifications), whereas CD3+ T-lymphocytes (Figure 6B,D,F different magnifications) did not reveal B19V DNA. This finding suggests that B19V preferentially infects B lymphocytes in the lymphoid tissue.

### 3.6. Detection of Virus Replication Using Sense and Antisense B19V DNA Probes via Non-Radioactive ISH

**Figure 7:**
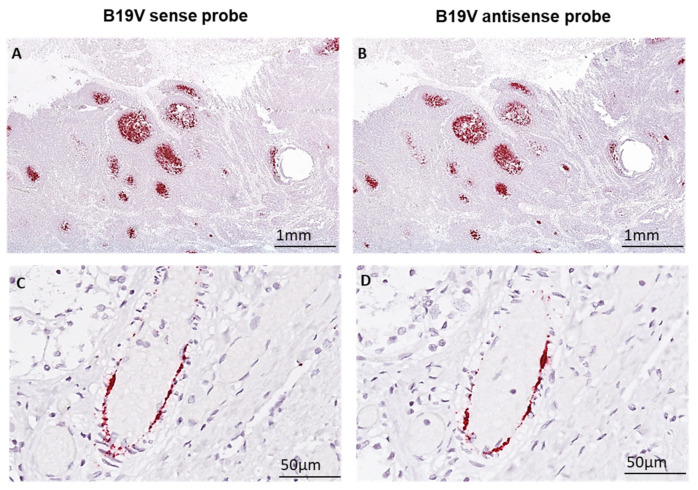
B19V DNA/mRNA is present in the germinal centres of secondary lymph follicles and in endothelial cells of small cardiac vessels. (**A**,**B**) B19V nucleic acids are detected in lymphatic tissue using sense (B19V sense) and anti-sense (B19V anti-sense) probes in consecutive tissue sections (red signals). (**C**,**D**) Corresponding localization of B19V nucleic acids in endothelial cells of small vessels using sense and anti-sense probes in consecutive heart tissue sections (red).

To investigate the distribution of sense (S) and antisense (AS) B19V nucleic acids in cardiac and splenic tissue, non-radioactive ISH was performed on consecutive tissue sections. Sense and antisense viral nucleic acids were detected in lymphocytes of splenic germinal centres of secondary lymphoid follicles (Figure 7A,B) as well as in the cardiac endothelial cells of small vessels (Figure 7C,D), visualizing replication of B19V. The patterns of viral sense and antisense nucleic acids, including B19V mRNAs, were identical in the spleen and heart.

## 4. Discussion

Lymphocytic myocarditis is a potentially life-threatening complication of B19V infection, especially in young children [17,18]. Although rare, this form of myocarditis can have serious consequences. Since January 2024, Germany has been experiencing an outbreak of B19V [19] (https://www.rki.de/DE/Content/Infekt/EpidBull/Archiv/2024/Ausgaben/24_24.pdf?__blob=publicationFile) (assessed on 16 July 2024), leading to a significant rise in severe lymphocytic myocarditis cases among young children as diagnosed in EMB. In the first seven months of 2024, we have already seen more than 50 cases of acute B19V-induced myocarditis in children; the vast majority of them are ≤ 2 years of age. Many of these children required mechanical heart support or even died due to the severity of their condition and present histological findings comparable to those of our two patients, A and B. This rise in B19V-associated myocarditis is particularly striking, considering the fact that in the recent coronavirus pandemic, we did not observe any acute cases of B19V myocarditis in children. This observation stresses the fact that social distancing measures and heightened public health interventions during the pandemic effectively prevented the transmission of B19V among this vulnerable population. Now, as these restrictions have been relaxed, increased social interactions obviously facilitate the spread of B19V, underlining the need for vigilant monitoring of potential myocarditis cases in young children.

Acute lymphocytic myocarditis was found to be associated with B19V infection in 46% of our cases. Cardiac virus load, but not the virus load in the blood, was found to correlate with the outcome of myocarditis, stressing the fact that the extent of cardiac viral infection/replication determines the intensity of lymphocytic inflammation in the heart. It is worth noting that our two autopsy cases of children (12 and 17 months old) with fulminant lymphocytic myocarditis showed a high B19V load in the heart, with up to 5.8 × 10^4^ copies/µg total isolated cardiac DNA. The reason for the higher B19V load in EMB of very young infants compared to older children might reflect differences in the activity of the immune system. It is known that deficits in T cell activation and function in neonates and young children are particularly seen in the CD4+ T cell compartment, where these cells reveal reduced levels of critical cytokines such as IFN-γ and interleukins including IL-2, IL-13, IL-5, and IL-17 [20]. This deficiency has been attributed to several factors, including reduced sensitivity to T cell receptor stimulation, increased apoptosis following proliferation, lack of available antigens for T cell priming, and inefficient stimulation by immature antigen-presenting cells [21]. These aspects highlight potential mechanistic failures that delay the maturation of CD4+ T cell activation and function, facilitating viral infection in younger children [20,22,23]. Whether these mechanisms play a role in the outcome of B19V infection in young children has to be determined in future investigations.

Another interesting aspect of our study was the finding that, in addition to endothelial cells, B lymphocytes in lymphatic tissue are productively infected. To our knowledge, our study is the first to report active replication of B19V in situ in B cells of lymphatic tissue in children with myocarditis. It is well known that viruses that target B cells can significantly impair the immune system via different mechanisms. For example, hepatitis C virus (HCV) can infect B cells, thereby disrupting B cell function and resulting in reduced antibody production [24]. Epstein–Barr virus (EBV), which infects B cells through the CD21 receptor, can develop mechanisms to evade immune surveillance by reducing the presentation of viral antigens on the cell surface [25]. This hinders the immune system’s ability to recognize and eliminate the virus, allowing EBV to persist in B cells. Persistent infection of B cells has also been demonstrated for B19V in tonsillar tissue [26]. B19V uptake into B cells was found to be antibody-mediated and CD32-dependent. The CD32 receptor was previously found to be important in the downregulation of B cell responses, and its deficiency or reduced expression correlates with prolonged humoral or autoimmune reactivity [27]. How productively infected B cells in lymphatic tissue influence immunity and, thus, the outcome of acute B19V myocarditis in our children remains to be determined.

In our study, we found that an acute systemic B19V infection induced inflammation only in the heart but not in other organs. We detected infection of endothelial cells in various organs, including the liver, kidneys, lungs, and spleen, but it was not associated with an inflammatory response. Interestingly, endothelial cells from different tissues and organs show a heterogeneous response to inflammatory stimuli and variability in gene expression [28]. Paik et al. identified tissue-specific endothelial cells that were able to adapt their phenotypes to the functional demands of each organ or condition [29]. We found that B19V replicates in endothelial cells of small arterioles and venules in the heart but not in capillaries. In particular, endothelial cells in capillaries have reduced levels of B19V-specific receptors, such as globoside, α5β1 integrin, and Ku80 [30,31,32,33]. Globoside is more abundant in the endothelium of arterioles and venules, facilitating B19V infection, while α5β1 integrin, a co-receptor for B19V, shows increased expression during inflammation [32]. Integrins, which are critical for cell adhesion and signal transduction, vary in expression among endothelial cells, impacting B19V infection efficiency [32,33]. Ku80, another co-receptor, aids B19V entry into the cell nucleus, with higher expression correlating with increased susceptibility to infection [27,31]. Capillaries, on the other hand, due to their role in nutrient and gas exchange and their exposure to lower pressure, exhibit less of these stress-induced features. Consequently, the expression of genes involved in responding to mechanical stress, including Ku80, is less pronounced. This aligns with the physiological role of capillaries in maintaining a stable environment for exchange processes rather than withstanding high mechanical loads [34]. This receptor heterogeneity may explain the missing B19V infection in the capillaries of the myocardium.

B19V non-structural protein (NS1) is known to control the production of the inflammatory cytokine interleukin 6 (IL-6). IL-6 induces immune cells and cardiomyocytes to express CD11b/CD18 and intercellular adhesion molecule-1 (ICAM-1) and chemokines, thereby initiating different inflammatory responses and consecutive damage to the heart [35,36]. Additionally, it has already been shown that the NS1 protein of B19V can induce cytotoxicity and apoptosis by activating the caspase 3 and 9 pathways through mitochondrial cell death [37,38]. Previously, it has shown that B19V infection of the heart is associated with cardiac inflammation and disturbances in microcirculation [1,4]. B19V infection of small vessels in the myocardium was found to impair microcirculation with the consequence of cardiac myocyte necrosis [5]. The exact mechanism through which B19V contributes to fatal virus-induced myocarditis is not known but is likely to involve several virus-associated signaling pathways. E.g., the VP1 unique region of B19V is known to induce myocardial injury by activating phosphorylated p-38 and NF-κB signaling [39]. On the other hand, NS1 can induce cell cycle arrest in the G2 phase by activating the ATR (Ataxia-Telangiectasia Mutated and Rad3-related) pathway, which in turn phosphorylates CDC25C and inactivates the cyclin B1/cyclin-dependent kinase 1 (CDK1) complex [40]. We have also seen other viral infections in myocarditis cases among children, such as HHV6 or EV, but these viruses infect other cardiac cell types and thus trigger different immune mechanisms not addressed in this study. Further investigations are required to investigate the suspected relationship between B19V and other viruses (especially HHV6, [41]) in the pathogenesis of myocarditis in children. Overall, this study sheds light on the critical role of B19V infection in mediating cardiac inflammatory responses in children. Especially in young children, the outcome of B19V myocarditis can be life-threatening and should be monitored very closely. It underscores the need for endomyocardial biopsy for the early and accurate diagnosis of viral myocarditis as a prerequisite for the appropriate treatment strategies. In particular, it has to be evaluated whether and when immunomodulatory agents, e.g., against IL-1, are useful to treat B19V myocarditis in children. Mechanistically, it is known that the B19V structural protein 1 region increases the production of interleukin-1 in macrophages, which are abundantly present in the hearts of our children already early in the infection [42]. Therapy with e.g., Interferon-ß [6] or Anakinra [43] for acute B19V-induced myocarditis might help to control systemic inflammation. It is hoped that our database of B19V myocarditis cases in children will be helpful in uncovering complexities in cellular landscapes and immune-mediated mechanisms in viral myocarditis, applying, e.g., single-cell omics and multiomics for high-resolution molecular profiling for a better understanding and treatment strategies of this disease.

## Figures and Tables

**Table 1:** Viral load of different organs from patients A and B.

	Patient A	Patient B
Heart	58,800	7250
Brain	3490	n.a.
Lung	31,000	8000
Lymph node	439,250	475,000
Spleen	729,000	370,000
Liver	17,000	2700
Kidney	7400	5800

B19V DNA load is given as the number of copies per µg of total isolated DNA from different organs in patients A and B. n.a.= not available.

## Data Availability

The original contributions presented in this study are included in the article; further inquiries can be directed to the corresponding author.

## References

[1] Bock C.T., Klingel K., Kandolf R. (2010). Human Parvovirus B19-Associated Myocarditis. N. Engl. J. Med..

[2] Broliden K., Tolfvenstam T., Norbeck O. (2006). Clinical Aspects of Parvovirus B19 Infection. J. Intern. Med..

[3] Young N.S., Brown K.E. (2004). Parvovirus B19. N. Engl. J. Med..

[4] Klingel K., Sauter M., Bock C.T., Szalay G., Schnorr J.J., Kandolf R. (2004). Molecular Pathology of Inflammatory Cardiomyopathy. Med. Microbiol. Immunol..

[5] Bultmann B.D., Klingel K., Sotlar K., Bock C.T., Baba H.A., Sauter M., Kandolf R. (2003). Fatal Parvovirus B19-Associated Myocarditis Clinically Mimicking Ischemic Heart Disease: An Endothelial Cell-Mediated Disease. Hum. Pathol..

[6] Esmel-Vilomara R., Dolader P., Izquierdo-Blasco J., Balcells J., Sorli M., Escudero F., Vera E., Gran F. (2022). Parvovirus B19 Myocarditis in Children: A Diagnostic and Therapeutic Approach. Eur. J. Pediatr..

[7] Simpson K.E., Storch G.A., Lee C.K., Ward K.E., Danon S., Simon C.M., Delaney J., Tong A., Canter C.E. (2016). High Frequency of Detection by PCR of Viral Nucleic Acid in the Blood of Infants Presenting with Clinical Myocarditis. Pediatr. Cardiol..

[8] Soderlund-Venermo M., Hokynar K., Nieminen J., Rautakorpi H., Hedman K. (2002). Persistence of Human Parvovirus B19 in Human Tissues. Pathol. Biol..

[9] Kuhl U., Pauschinger M., Seeberg B., Lassner D., Noutsias D., Poller W., Schultheiss H.P. (2005). Viral Persistence in the Myocardium is Associated with Progressive Cardiac Dysfunction. Circulation.

[10] Tschope C., Bock C.T., Kasner M., Noutsias M., Westermann D., Schwimmbeck P.L., Pauschinger M., Poller W.C., Kuhl U., Kandolf R. (2005). High Prevalence of Cardiac Parvovirus B19 Infection in Patients with Isolated Left Ventricular Diastolic Dysfunction. Circulation.

[11] Caforio A.L., Pankuweit S., Arbustini E., Basso C., Gimeno-Blanes J., Felix S.B., Fu M., Helio T., Heymans S., Jahns R. (2013). Current State of Knowledge on Aetiology, Diagnosis, Management, and Therapy of Myocarditis: A Position Statement of the European Society of Cardiology Working Group on Myocardial and Pericardial Diseases. Eur. Heart J..

[12] Ho H.T., Peischard S., Strutz-Seebohm N., Seebohm G. (2021). Virus-Host Interactions of Enteroviruses and Parvovirus B19 in Myocarditis. Cell. Physiol. Biochem..

[13] Brown K.E., Young N.S. (1995). Parvovirus B19 Infection and Hematopoiesis. Blood. Rev..

[14] Aretz H.T. (1987). Myocarditis: The Dallas Criteria. Hum. Pathol..

[15] Greulich S., Kindermann I., Schumm J., Perne A., Birkmeier S., Grun S., Ong P., Schaufele T., Klingel K., Schneider S. (2016). Predictors of Outcome in Patients with Parvovirus B19 Positive Endomyocardial Biopsy. Clin. Res. Cardiol..

[16] Klingel K., Stephan S., Sauter M., Zell R., McManus B.M., Bultmann B., Kandolf R. (1996). Pathogenesis of Murine Enterovirus Myocarditis: Virus Dissemination and Immune Cell Targets. J. Virol..

[17] Molina K.M., Garcia X., Denfield S.W., Fan Y., Morrow W.R., Towbin J.A., Frazier E.A., Nelson D.P. (2013). Parvovirus B19 Myocarditis Causes Significant Morbidity and Mortality in Children. Pediatr. Cardiol..

[18] Nigro G., Bastianon V., Colloridi V., Ventriglia F., Gallo P., D’Amati G., Koch W.C., Adler S.P. (2000). Human Parvovirus B19 Infection in Infancy Associated with Acute and Chronic Lymphocytic Myocarditis and High Cytokine Levels: Report of 3 Cases and Review. Clin. Infect. Dis..

[19] Beck R., Exler S., Enders M. (2024). Parvovirus B19-Infektion und Schwangerschaft. Epidemiol. Bull..

[20] Adkins B., Leclerc C., Marshall-Clarke S. (2004). Neonatal Adaptive Immunity Comes of Age. Nat. Rev. Immunol..

[21] Sharma S.K., Pichichero M.E. (2013). Deficiencies in the Cd4(+) T-Helper Cell Arm of the Immune System of Neonates and Young Children. Pediatr. Allergy Immunol. Pulmonol..

[22] Hassan J., Reen D.J. (1996). Reduced Primary Antigen-Specific T-Cell Precursor Frequencies in Neonates is Associated with Deficient Interleukin-2 Production. Immunology.

[23] Zaghouani H., Hoeman C.M., Adkins B. (2009). Neonatal Immunity: Faulty T-Helpers and the Shortcomings of Dendritic Cells. Trends Immunol..

[24] Desombere I., Van Houtte F., Farhoudi A., Verhoye L., Buysschaert C., Gijbels Y., Couvent S., Swinnen W., Van Vlierberghe H., Elewaut A. (2021). A Role for B Cells to Transmit Hepatitis C Virus Infection. Front. Immunol..

[25] Silva J.M., Alves C.E.C., Pontes G.S. (2024). Epstein-Barr Virus: The Mastermind of Immune Chaos. Front. Immunol..

[26] Pyoria L., Toppinen M., Mantyla E., Hedman L., Aaltonen L.M., Vihinen-Ranta M., Ilmarinen T., Soderlund-Venermo M., Hedman K., Perdomo M.F. (2017). Extinct Type of Human Parvovirus B19 Persists in Tonsillar B Cells. Nat. Commun..

[27] von Kietzell K., Pozzuto T., Heilbronn R., Grossl T., Fechner H., Weger S. (2014). Antibody-Mediated Enhancement of Parvovirus B19 Uptake into Endothelial Cells Mediated by a Receptor for Complement Factor C1q. J. Virol..

[28] Immanuel J., Yun S. (2023). Vascular Inflammatory Diseases and Endothelial Phenotypes. Cells.

[29] Paik D.T., Tian L., Williams I.M., Rhee S., Zhang H., Liu C., Mishra R., Wu S.M., Red-Horse K., Wu J.C. (2020). Single-Cell Rna Sequencing Unveils Unique Transcriptomic Signatures of Organ-Specific Endothelial Cells. Circulation.

[30] Wang W., Jia H., Hua X., Song J. (2024). New Insights Gained from Cellular Landscape Changes in Myocarditis and Inflammatory Cardiomyopathy. Heart Fail. Rev..

[31] Munakata Y., Saito-Ito T., Kumura-Ishii K., Huang J., Kodera T., Ishii T., Hirabayashi Y., Koyanagi Y., Sasaki T. (2005). Ku80 Autoantigen as a Cellular Coreceptor for Human Parvovirus B19 Infection. Blood.

[32] Mrugacz M., Bryl A., Falkowski M., Zorena K. (2021). Integrins: An Important Link between Angiogenesis, Inflammation and Eye Diseases. Cells.

[33] Steiger K., Quigley N.G., Groll T., Richter F., Zierke M.A., Beer A.J., Weichert W., Schwaiger M., Kossatz S., Notni J. (2021). There is a World Beyond Alphavbeta3-Integrin: Multimeric Ligands for Imaging of the Integrin Subtypes Alphavbeta6, Alphavbeta8, Alphavbeta3, and Alpha5beta1 by Positron Emission Tomography. EJNMMI Res..

[34] Anwar M.A., Shalhoub J., Lim C.S., Gohel M.S., Davies A.H. (2012). The Effect of Pressure-Induced Mechanical Stretch on Vascular Wall Differential Gene Expression. J. Vasc. Res..

[35] Sol N., Le Junter J., Vassias I., Freyssinier J.M., Thomas A., Prigent A.F., Rudkin B.B., Fichelson S., Morinet F. (1999). Possible Interactions between the Ns-1 Protein and Tumor Necrosis Factor Alpha Pathways in Erythroid Cell Apoptosis Induced by Human Parvovirus B19. J. Virol..

[36] Gwechenberger M., Mendoza L.H., Youker K.A., Frangogiannis N.G., Smith C.W., Michael L.H., Entman M.L. (1999). Cardiac Myocytes Produce Interleukin-6 in Culture and in Viable Border Zone of Reperfused Infarctions. Circulation.

[37] Poole B.D., Kivovich V., Gilbert L., Naides S.J. (2011). Parvovirus B19 Nonstructural Protein-Induced Damage of Cellular DNA and Resultant Apoptosis. Int. J. Med. Sci..

[38] Hsu T.C., Wu W.J., Chen M.C., Tsay G.J. (2004). Human Parvovirus B19 Non-Structural Protein (Ns1) Induces Apoptosis through Mitochondria Cell Death Pathway in Cos-7 Cells. Scand. J. Infect. Dis..

[39] Pankuweit S., Klingel K. (2013). Viral Myocarditis: From Experimental Models to Molecular Diagnosis in Patients. Heart Fail. Rev..

[40] Xu P., Zhou Z., Xiong M., Zou W., Deng X., Ganaie S.S., Kleiboeker S., Peng J., Liu K., Wang S. (2017). Parvovirus B19 Ns1 Protein Induces Cell Cycle Arrest at G2-Phase by Activating the Atr-Cdc25c-Cdk1 Pathway. PLoS Pathog..

[41] Bock C.T., Duchting A., Utta F., Brunner E., Sy B.T., Klingel K., Lang F., Gawaz M., Felix S.B., Kandolf R. (2014). Molecular Phenotypes of Human Parvovirus B19 in Patients with Myocarditis. World. J. Cardiol..

[42] Tzang B.S., Chiu C.C., Tsai C.C., Lee Y.J., Lu I.J., Shi J.Y., Hsu T.C. (2009). Effects of Human Parvovirus B19 Vp1 Unique Region Protein on Macrophage Responses. J. Biomed. Sci..

[43] Butin M., Mekki Y., Phan A., Billaud G., Di Filippo S., Javouhey E., Cochat P., Belot A. (2013). Successful Immunotherapy in Life-Threatening Parvovirus B19 Infection in a Child. Pediatr. Infect. Dis. J..

